# Smoking at time of CIS increases the risk of clinically definite multiple sclerosis

**DOI:** 10.1007/s00415-018-8780-4

**Published:** 2018-02-20

**Authors:** Roos M. van der Vuurst de Vries, Julia Y. Mescheriakova, Tessel F. Runia, Theodora A. M. Siepman, Beatrijs H. A. Wokke, Johnny P. A. Samijn, Rogier Q. Hintzen

**Affiliations:** 1 Department of Neurology, MS Centre ErasMS, Erasmus MC, P.O. Box 2040, 3000 CA Rotterdam, The Netherlands; 2 Department of Neurology, Maasstad Hospital, Rotterdam, The Netherlands

**Keywords:** Clinically isolated syndrome, Multiple sclerosis, Smoking

## Abstract

**Background:**

Cigarette smoking is a modifiable risk factor that influences the disease course of patients with multiple sclerosis (MS). However, in patients with a clinically isolated syndrome (CIS), there are conflicting results about the association between smoking and the risk of a subsequent MS diagnosis. The aim of this study was to determine the risk of clinically definite MS (CDMS) in smoking and non-smoking patients at time of a first demyelinating event.

**Methods:**

Two hundred and fifty patients, aged 18–50 years, were included in our prospective CIS cohort. At time of the first neurological symptoms, patients completed a questionnaire about smoking habits. Cox regression analyses were performed to calculate univariate and multivariate hazard ratios for CDMS diagnosis in smoking and non-smoking CIS patients.

**Results:**

One hundred and fourteen (46%) CIS patients were diagnosed with CDMS during a mean follow-up of 58 months. In total, 79 (32%) patients smoked at time of CIS. Sixty-seven % of the smoking CIS patients were diagnosed with CDMS during follow-up compared to 36% of the non-smoking CIS patients (*p* < 0.001). Smoking at time of CIS was an independent predictor for CDMS diagnosis (HR 2.3; *p* = 0.002). Non-smoking CIS patients who had a history of smoking did not have a higher risk for CDMS than those who had never smoked.

**Conclusions:**

Smoking at time of CIS was an independent risk factor for a future CDMS diagnosis. This is an additional argument to quit smoking at time of the first attack of suspected MS.

## Introduction

Multiple sclerosis (MS) is a chronic inflammatory autoimmune disease, influenced by environmental factors in genetically susceptible individuals [1]. This results in demyelination, axonal loss, and neurodegeneration [1–3]. The course of MS is heterogeneous in severity and prognosis [1].

One of the environmental factors influencing the course of MS is cigarette smoking [4]. Studies in MS patients and healthy controls consistently provide evidence that both active and passive smoking result in an increased risk of MS and disease progression [4–7]. Smoking does not only increase MS risk, it also shortens the time to the secondary progressive phase of MS (SPMS) [8, 9]. It has been shown that after cessation of smoking, negative effects slowly decrease, independent of the cumulative dose of smoking [8, 10].

Studies show that the risk of MS associated with HLA genotypes is influenced by smoking. This interaction leads to a much stronger effect on MS risk than the cumulative effect of genetic risk factors and smoking together [11, 12].

In the majority of cases (85–90%), MS starts as a clinically isolated syndrome (CIS), followed by novel episodes of neurological symptoms resulting from inflammation of the central nervous system (CNS). However, a CIS attack can also remain a single event [13].

Only a few studies are available in CIS patients that investigated the effect of smoking on subsequent MS risk. Firm conclusions in these studies are hampered by several methodologic issues such as low patient numbers, retrospective designs [14–16], or considerable numbers of CIS patients treated with interferon beta [17, 18]. On the other hand, several studies in MS patients do suggest a link between smoking and MS progression [7]. Therefore, we determined the effect of smoking on clinically definite MS (CDMS) risk in a large prospective cohort of predominantly untreated CIS patients. It is important to know if smoking is associated with a future MS diagnosis, as smoking is up to now, one of the few modifiable risk factors for disease progression in MS.

## Methods

### Patients

Data were collected prospectively from patients with CIS at the Neurology Department of Erasmus MC University Hospital in Rotterdam, a tertiary referral centre for patients with MS. Data collection was in collaboration with several regional hospitals in The Netherlands. Included patients had their first symptoms between May 2006 and June 2017. Patients were aged between 18 and 50 years, with no history of previous neurological symptoms suggestive of CNS demyelination. CIS patients were included within 6 months following the first neurological symptoms. Patients with alternative diagnoses were excluded from the analyses. At baseline, a magnetic resonance imaging (MRI) scan and routine laboratory tests were utilized to rule out alternative diagnoses [19]. Following inclusion, patients were reassessed at least annually at the Neurological Outpatient Department.

### Questionnaire

At baseline, CIS patients completed a questionnaire to gather information about smoking habits, including when they first started smoking, non- or reduced-smoking periods and how many cigarettes were smoked within these periods. Using results of the questionnaire, we were able to calculate the pack-years per patient.

### Standard protocol approvals, and patient consent

This study was approved by the Medical Ethics Committee of Erasmus MC Rotterdam. Written informed consent was obtained from all patients.

### Definitions

A relapse was defined as new symptoms or subacute worsening of existing symptoms after 30 days of improvement, or stable disease and no evidence of alternative diagnosis. Symptoms had to exist longer than 24 h and not to be preceded by fever [20]. All exacerbations were confirmed by neurological examination. CDMS was defined as clinical dissemination in space and time with two exacerbations and (para) clinical evidence of two separate lesions, as described by Poser et al. [21]. Patients who were diagnosed with CDMS during follow-up are referred to as CIS–CDMS and patients who remained CIS are referred to as CIS–CIS. Expanded Disability Status Scale (EDSS) scores were performed annually when patients were diagnosed with CDMS [22]. EDSS performed within 3 months after a relapse were not used in the analyses. Follow-up was calculated by subtracting CIS date from the last visit date. Patients were defined as smokers when they were smoking regularly at time of CIS. Non-smokers were those who did not smoke at time of CIS. Patients were defined as ex-smokers when they were not smoking at time of CIS, but did have a history of smoking in the years prior to CIS. To calculate pack-years, the number of years smoked was multiplied by the number of cigarettes smoked per day/20 in that period.

### Statistical analysis

Statistical analyses were done using SPSS, version 21.0 (SPSS Inc) for Windows and GraphPad Prism5 (GraphPad) for Windows. Nominal data comparison between groups was done using Chi-square or Fisher’s exact test [gender, type of clinical onset, oligoclonal bands (OCB), ≥ 9 T2 lesions on baseline MRI, disease modifying therapy (DMT) at time of CIS, smoking at time of CIS, SPMS, and alcohol use]. The Kolmogorov–Smirnov test was performed to assess normality of data distribution. To compare continuous data, we applied a two-tailed *t* test (age at onset and follow-up time) or when the data were non-parametric, a Mann–Whitney *U* test (time from CIS to CDMS and pack-years). Time to second attack was calculated from onset of the first symptoms. Cox-proportional hazard regression analyses were used to calculate univariate and multivariate hazard ratios (HR). Patients who did not have a second attack during follow-up were considered as censored observations. Hazard ratios were also obtained for time to EDSS 4.0 and time to EDSS 6.0. *p* values less than 0.05 were considered significant.

## Results

### Patient characteristics

We included 250 patients who completed the baseline questionnaire about smoking at time of CIS. Out of these 250 CIS patients, 114 (46%) patients had a second relapse and were diagnosed with CDMS during a mean follow-up time of 58.1 months (SD 35.9).

The median time (interquartile range; IQR) from CIS to CDMS was 23.3 months (8.9–44.3). The median time (IQR) between the first neurological symptoms and inclusion in the study was 1.2 months (0.3–2.9 months).

Fifty-seven (23%) patients who were not yet diagnosed with CDMS were treated with DMT. The patient characteristics are shown in Table 1.

**Table 1:** Patient characteristics (CIS–CDMS vs CIS–CIS patients)

	CIS patients (*n* = 250)	CIS–CDMS (*n* = 114)	CIS–CIS (*n* = 136)	*p* value^a^
Female sex, no. (%)	189 (75.6)	92 (80.7)	97 (71.3)	0.09
Age, mean (SD), years	33.6 (8.3)	32.6 (7.9)	34.5 (8.5)	0.07
Follow-up time, mean (SD), months	58.1 (35.9)	72.3 (30.4)	46.3 (36.0)	**<** **0.01**
Type of clinical onset, no. (%)				
Optic nerve	88 (35.2)	33 (28.9)	55 (40.4)	0.06
Spinal cord	90 (36.0)	45 (39.5)	45 (33.1)	0.30
Other localization	72 (28.8)	36 (31.6)	36 (26.5)	0.37
OCB, (> 1 band), (%)	113 (73.9)	63 (81.8)	50 (65.8)	**0.02**
≥ 9 lesions on T2-weighted images, no. (%)	96 (38.6)	53 (46.9)	43 (31.6)	**0.01**
DMT at time of CIS, no. (%)	57 (22.8)	26 (22.8)	31 (22.8)	1.00
Smoking at time of CIS, no. (%)	79 (31.6)	53 (46.5)	26 (19.1)	**<** **0.01**
Pack-years at time of CIS, median (IQR)	1.0 (0.0–5.9)	2.4 (0.0–9.5)	0.0 (0.0–2.7)	**<** **0.01**

*CIS* clinically isolated syndrome, *CIS–CDMS* patients who are diagnosed with CDMS during follow-up after CIS defined by Poser criteria, *CIS–CIS* not diagnosed with CDMS, *na* not applicable, *OCB* oligoclonal bands

^a^*P* value calculated between CIS–CDMS and CIS–CIS

### Smokers vs non-smokers

**Fig. 1:**
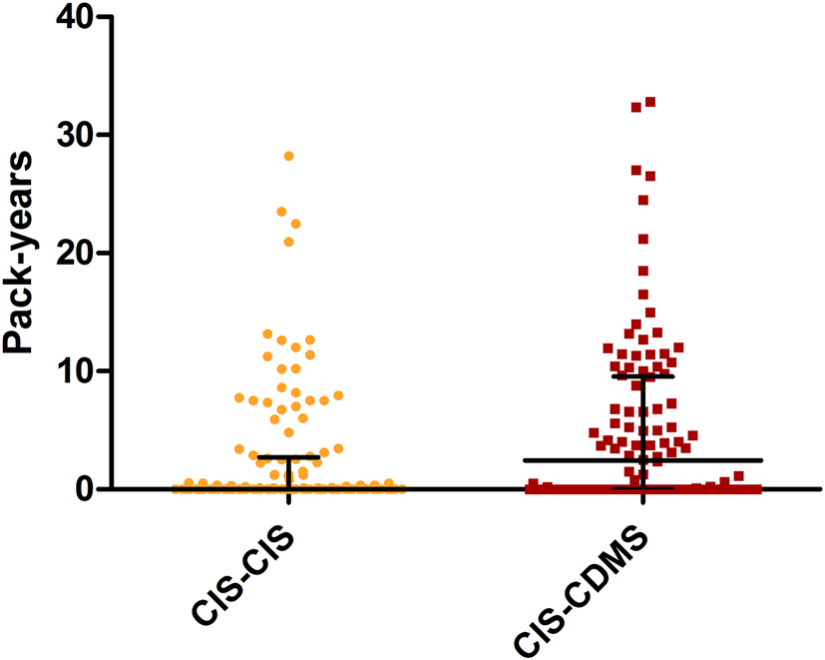
Pack-years in CIS patients. Comparison of pack-years between CIS–CIS and CIS–CDMS patients. Horizontal lines and error bars indicate median and IQR

In total, 79 out of 250 (32%) patients smoked at time of CIS. Fifty-three out of 79 (67%) smoking CIS patients were diagnosed with CDMS during follow-up compared to 61 out of 171 (36%) in the non-smoking CIS patients (*p* < 0.001). The number of pack-years was higher in the group that was diagnosed with CDMS (CIS–CDMS) during follow-up than in the group that remained CIS (CIS–CIS) (median (IQR) CIS–CDMS vs CIS–CIS: 2.4 (0.0–11.9) vs 0.0 (0.0–2.7) *p* = 0.004) (Fig. 1).

There were no differences between smokers and non-smokers in gender, localisation of first symptoms, age, OCB in CSF or MRI characteristics at baseline. Table 2 shows patient characteristics for smokers and non-smokers.

**Table 2:** Patient characteristics (smoking vs non-smoking CIS patients)

	Smoking CIS patients (*n* = 79)	Non-smoking CIS patients (*n* = 171)	*p* value
Female sex, no. (%)	58 (73.4)	131 (76.6)	0.59
Age, mean (SD), years	33.9 (7.7)	33.5 (8.5)	0.67
Follow-up time, mean (SD), months	60.7 (30.7)	57.0 (38.1)	0.45
Type of clinical onset, no. (%)			
Optic nerve	25 (31.6)	63 (36.8)	0.42
Spinal cord	29 (36.7)	61 (35.7)	0.87
Other localization	25 (31.6)	47 (26.3)	0.50
OCB, (> 1 band), (%)	43 (75.4)	70 (72.9)	0.73
≥ 9 lesions on T2-weighted images, no. (%)	33 (42.3)	63 (36.8)	0.41
DMT at time of CIS, no. (%)	24 (30.4)	33 (19.3)	**0.05**
CDMS, no. (%)	53 (67.1)	61 (35.7)	**<** **0.01**
SPMS, no. (%)	3 (3.8)	5 (2.9)	0.72
Alcohol use, no. of patients (%)	55 (69.6)	79 (46.2)	**<** **0.01**

*CIS* clinically isolated syndrome, *OCB* oligoclonal bands

### Association of smoking at time of CIS with a shorter time to CDMS

**Fig. 2:**
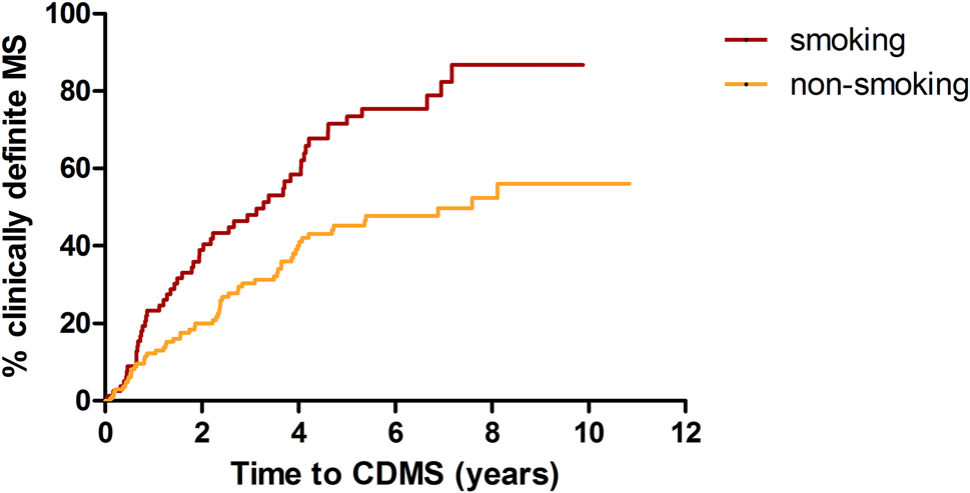
Time from CIS to CDMS in smoking and non-smoking patients. Kaplan–Meier curve for time from CIS to CDMS for smoking and non-smoking patients at time of CIS [log-rank test *p* < 0.001)]

Patients who smoked at time of CIS had a shorter time to CDMS diagnosis than patients who were not active smokers (univariate hazard ratio; HR 2.1 *p* < 0.001) (Fig. 2). Corrections were applied for multiple variables that are associated with a second attack (OCB in CSF, more than nine T2 lesions, gadolinium enhancing lesions on baseline MRI, and optic neuritis as first symptom, no DMT before CDMS). After these adjustments, multivariate COX regression analysis showed smoking as an independent predictor for a second attack. The HR was 2.3 (*p* = 0.002).

In a sub-analysis, we excluded the 50 CIS patients who had less than 2 years of follow-up. After exclusion, the results remained the same, HR 2.0 (*p* < 0.001).

Fifty-seven (23%) of the patients received DMT before CDMS diagnosis. When we excluded these patients, the HR was unchanged 2.0 (*p* = 0.001).

### Smoking in the past

In the group of patients that did not smoke at time of CIS (*n* = 171), 63 (37%) patients had a history of smoking in the past (ex-smokers). Smoking in the past did not predict CDMS diagnosis in the group of non-smoking CIS patients (HR 0.64, *p* = 0.12). Furthermore, in this non-smoking group (*n* = 171), the number of pack-years was not correlated with time to CDMS [HR per pack-years: 0.96 (*p* = 0.31)].

### Smoking at time of CIS and disability later in the disease

In this cohort, we collected EDSS data from 96/114 (84%) patients who were diagnosed with CDMS. Nineteen patients reached an EDSS of 4.0 and eight patients an EDSS of 6.0 during follow-up. Six out of these eight patients who reached an EDSS score of 6.0 or more were smoking at time of CIS. The HRs for both EDSS scores were not significant [HR for EDSS 4.0: 1.9 (*p* = 0.18) and HR for EDSS 6.0: 4.1 (*p* = 0.09)]. However, there was a trend towards faster disability progression in CDMS patients who were smoking at time of CIS.

## Discussion

In this prospective study of patients included after a first attack of suspected MS, we determined the risk of CDMS in a cohort of 250, mostly untreated smoking and non-smoking CIS patients. We demonstrated that smoking at time of CIS is associated with a shorter time to a second clinical attack and, therefore, an earlier diagnosis of CDMS.

Smoking is a well-established risk factor for MS and disease progression after MS diagnosis [4, 5]. This association is also found in other auto-inflammatory diseases such as rheumatoid arthritis (RA) and systemic lupus erythematosus (SLE) [23, 24].

Studies investigating the influence of smoking on MS risk in first attack patients have remained inconclusive, as described recently in a systematic review [25]. Three studies were relatively small and used retrospective data [14–16]. Other studies were large, but patients were treated with interferon beta immediately after CIS or 2 years after CIS, [17, 18]. Interferon beta treatment could have postponed MS diagnosis [26]. Therefore, the potential correlation between smoking and MS risk may be overshadowed by this disease-postponing therapy.

Compared to these studies, the present study has less confounding factors, it has a prospective design and only a small proportion of patients was treated with DMT before CDMS diagnosis.

In the multivariate analysis, we corrected our results for currently known predictors for CDMS diagnosis (large number of T2 lesions, contrast enhancing lesions on baseline MRI, unique OCB in CSF and localization of CIS) and for DMT before CDMS diagnosis [27]. After these corrections, smoking remained clearly predictive for CDMS diagnosis. Therefore, smoking status can potentially improve prediction of a future CDMS diagnosis in CIS patients. Accurately predicting CDMS diagnosis is important to prevent unnecessary treatment of patients with low disease activity [28].

The fact that smoking in the past in current non-smoking CIS patients was not associated with CDMS suggests that the harmful effects of smoking are reversible. This supports results of earlier studies, showing that after cessation of smoking, the negative effect on disability progression slowly decreases, independent to the cumulative dose of smoking [8, 10].

Our study has some limitations. Although the mean follow-up time was long (almost 5 years), there was a wide range. To overcome this, we used a COX regression model to correct for follow-up time. We also performed a sub-analysis, where CIS patients with less than 2 years of follow-up were excluded. Excluding these patients left 200 patients for analysis and did not change our results. Yet, for demonstration of an association between smoking at time of CIS and later disability (EDSS) a longer follow-up would be needed.

Second, there is a possibility that our results are explained by potential confounding lifestyle factors such as body mass index (BMI) or alcohol use. It has been shown that obesity is a risk factor for MS [29]. However, it is not likely that a high BMI explained the effect seen here, as obesity is more common in non-smokers [30]. A Swedish study showed an inverse association of alcohol consumption with MS [31]. We did not observe an effect of alcohol use on CDMS diagnosis in the regression analysis (data not shown).

Third, a follow-up MRI scan was not performed according to a fixed protocol. Instead, we used the classic Poser criteria that are based on clinical manifestations to define CDMS. Thus, we can only claim an association with clinical disease activity but not with lesion accrual on MRI scan.

It is not likely that CIS patients with a second attack during follow-up had over-reported smoking at time of CIS. Even in case recall would play a serious role here, recall of smoking would be expectedly more strong for the question of past smoking [32]. Yet, it was only recent smoking, more plausibly related to concurrent biological processes just before, during and after the first demyelinating attack, that showed an association.

The exact influence of ongoing smoking on the progression of the auto-inflammatory process around a first clinical attack of demyelination remains to be determined. It may involve several pathways, including both direct and indirect influences of tobacco toxins and smoke particles on T cells and antigen presenting cells [33].

To conclude, we show in a large prospective cohort of CIS patients that smoking at time of CIS is an independent risk factor for a future CDMS diagnosis. Smoking status could even be a relevant parameter in predictive models on a possible MS disease course after CIS. Since smoking is a modifiable risk factor, our study draws attention to the relevance of counselling patients about smoking. Though intervention studies will be difficult to execute, this study may provide evidence for the argument to quit smoking for patients with a first attack of suspected MS.
